# Exploring Photobiomodulation as a Potential Novel Intervention for Developmental Stuttering: A Review and Hypothesis

**DOI:** 10.3390/jcm15052041

**Published:** 2026-03-07

**Authors:** Borja Ignacio Ferreras, Manuela Goyeneche, Paolo Cassano, Frank H. Guenther, Victoria Tumanova

**Affiliations:** 1 Division of Neuropsychiatry and Neuromodulation, Massachusetts General Hospital, Boston, MA 02114, USA; 2 Department of Psychiatry, Harvard Medical School, Boston, MA 02115, USA; 3 Faculty of Medicine, Pontificia Universidad Javeriana, Bogota D.C., Bogota 110231, Colombia; 4 Department of Speech, Language & Hearing Sciences, Boston University, Boston, MA 02215, USA; 5 Department of Communication Sciences and Disorders, Syracuse University, Syracuse, NY 13244, USA

**Keywords:** low-level light therapy, developmental stuttering, speech disorders, transcranial photobiomodulation

## Abstract

Developmental stuttering (DS) is a complex neurodevelopmental disorder affecting approximately 5% of children, characterized by involuntary disruptions in speech fluency. Despite its prevalence, the precise pathophysiology remains elusive, and current behavioral and pharmacological interventions often yield variable long-term efficacy. This scoping review evaluates the therapeutic potential of transcranial photobiomodulation (t-PBM), a non-invasive neuromodulation technique, by summarizing its mechanisms of action with the known neurophysiological deficits of DS. Evidence indicates that DS is associated with reduced regional cerebral blood flow (rCBF) in Broca’s area, mitochondrial dysfunction, and impaired neural connectivity. t-PBM may address these deficits by stimulating cytochrome c oxidase, thereby increasing ATP production and triggering nitric oxide-mediated vasodilation to enhance rCBF. Furthermore, t-PBM promotes neuroplasticity, modulates astrocyte function—potentially counteracting GNPTAB-related deficits—and exhibits anxiolytic effects that may alleviate the secondary psychological burden of DS. By targeting these multifactorial underpinnings, t-PBM may represent a promising, low-risk adjunct or primary intervention for DS, though this remains to be tested empirically. While the theoretical framework is robust, clinical trials are needed to determine whether t-PBM has therapeutic utility, to optimize treatment parameters, establish longitudinal efficacy, and explore synergistic effects with established speech-language therapies.

## 1. Introduction

Developmental stuttering (DS) is a neurodevelopmental speech disorder characterized by the disturbance of the fluency (i.e., the naturally timed “flow”) of speech [1]. It manifests as blocks, prolongations, or repetitions of sounds in speech, typically at the beginning of utterances. Moments of stuttering can also be associated with non-speech secondary behaviors such as orofacial muscle spasms (rapid eye blinks, tremors of the lips or jaw, facial tics, head jerks, etc.) [2,3]. DS affects up to 5% of the pediatric population, on average, between the ages of 2.5 and 5 years, with 75 to 90% of children recovering spontaneously, without formal therapy. The remaining 25–10% develop chronic stuttering and may seek treatment, setting an approximate 1% for the total adult population with chronic DS. Some well-documented risk factors include male sex and family history, with others including twin birth, adverse perinatal outcomes, and birth-associated trauma [2,3]. Acquired forms of stuttering are usually secondary to brain damage or neurodegenerative diseases but can also be psychogenic.

DS has been associated with a significantly worsened quality of life, a psychosocial burden, and a higher incidence of anxiety and depression [1,4]. A recent study revealed that adults who stutter tend to focus their attention on negative thoughts about stuttering and its consequences, also reporting feelings of shame, embarrassment, fear, helplessness, isolation, and frustration [5].

The origin and causes of DS have been studied for over a century, with initial studies dating back to 1841.

Currently, interventions can be grouped into three main categories. First, behavioral intervention therapies, such as speech therapy or cognitive-behavioral therapy, are evidence-based and have been shown to be effective; however, they are not without limitations. These approaches require highly trained/specialized personnel, may not be covered by health insurance, are highly time-sensitive, and are more oriented towards teaching coping strategies, rather than improving speech fluency. Psychopharmacological therapy, including Dopamine Antagonists, VMAT-2 inhibitors, and GABA modulators, has been trialed for DS [6]. These options have minimal impact on stuttering, and they expose patients to many unwanted side effects, may cause interactions with other drugs, and are not FDA-approved for stuttering. Other antidepressants and anxiolytics have been tested, though results indicate a reduction in anxiety or other mood symptoms, rather than a positive impact on speech fluency, speed, or rhythm. Finally, a small number of studies have reported the impact on stuttering of emerging technological interventions such as Transcranial Direct Current Stimulation (tDCS), Repetitive Transcranial Magnetic Stimulation (rTMS), and Deep Brain Stimulation (DBS), showing promising results in small test groups [3,7,8,9]. These preliminary findings call for larger studies that would establish the positive effects of these interventions on DS, including any long-term benefits, and explore any adverse effects of these therapies. These last treatment options also present high costs associated with equipment and treatment sessions, limited availability and accessibility, and the need for highly trained personnel.

Transcranial photobiomodulation (t-PBM) is an emerging therapy that uses infrared light to modulate brain activity, promote neuroprotection and healing, reduce inflammation, and improve cognitive function [10]. It is noninvasive and highly tolerable, and it has successfully been applied to treat depression, anxiety, traumatic brain injury, autism disorder, and Alzheimer’s disease, although it is not FDA-approved.

This article is structured as a dual-purpose review. First, it provides a focused scoping review of the established pathophysiological mechanism underlying DS, synthesizing findings from neuroimaging, genetic, and clinical studies. Second, building on this foundation, it presents a hypothesis-driven exploration of t-PBM as a potential therapeutic intervention for DS. By systematically mapping the known effects of t-PBM onto the multifactorial deficits implicated in DS-inducing reduced cerebral blood flow, metabolic dysfunction, and impaired neuroplasticity, we aim to construct a scientifically grounded framework to guide and stimulate future empirical investigation. It is important to note that this synthesis is intended to generate hypotheses for preclinical and clinical research, not to assert current clinical efficacy.

## 2. Methods

Initially, a search equation was designed and applied to ScienceDirect, SciELO, Google Scholar, Scopus, PubMed, and Web of Science to identify previous studies on t-PBM in stuttering; however, no relevant results were found. The search terms included were: “Photobiomodulation” OR “Light Medicine” OR “Low Level Laser Therapy” AND “Stuttering” OR “Stammering”.

To improve transparency of the selection process, inclusion and exclusion criteria were applied during study identification. Studies were included if they (1) investigated neurobiological, genetic, physiological, or developmental mechanisms of developmental stuttering using human or relevant animal data; or (2) evaluated the effects of photobiomodulation on cerebral blood flow, neuronal metabolism, neuroplasticity, astrocyte biology, brain injury, or mood-related pathways relevant to the proposed pathophysiology of stuttering. Priority was given to peer-reviewed original studies and systematic or narrative reviews with direct mechanistic or clinical relevance.

Studies were excluded if they focused exclusively on behavioral or speech-therapy outcomes without neurobiological correlates, addressed acquired or neurogenic stuttering without developmental relevance, or investigated photobiomodulation effects outside the central nervous system or neuropsychiatric domains.

The final set of 35 included articles reflects a targeted selection, driven by the proposed hypothesis, intended to map convergent pathophysiological mechanisms of developmental stuttering with established biological effects of photobiomodulation. Given the narrative and integrative nature of this review, formal systematic review procedures (e.g., PRISMA flow diagram or risk-of-bias scoring) were not applied. This approach introduces potential selection bias, including emphasis on mechanistic overlap and underrepresentation of null or conflicting findings; therefore, interpretations should be considered hypothesis-generating rather than confirmatory.

## 3. Pathophysiology of Stuttering

The following subsections synthesize the current understanding of the neurobiological, genetic, and physiological mechanisms underlying developmental stuttering (DS). It is important to distinguish between findings that are well established through replicated studies (Section 3.1, Section 3.2 and Section 3.3) and those that remain more speculative or are derived from preliminary evidence (Section 3.4, Section 3.5 and Section 3.6).

### 3.1. Cerebral Blood Flow at Rest

Several studies have found a link between stuttering and reduced regional cerebral blood flow (rCBF) measured at rest. Using xenon-133 single-photon emission computed tomography, Pool et al. (1991) [11] studied 20 adult stutterers (ages 24 to 59 years), compared them to 78 age-matched controls, and found evidence of reduced global, absolute flow as well as anomalous flow asymmetries in the anterior cingulate gyrus, superior and middle temporal gyri, and left inferior frontal gyrus (IFG). Desai et al. (2017) [12] used pulsed arterial spin labeling magnetic resonance imaging to investigate rCBF in 26 patients with developmental stuttering (ages 5–59 years) and 36 healthy controls (ages 6–50 years). They identified lower rCBF at rest in both children and adults who stutter in bilateral IFG and the superior frontal gyrus (SFG). Furthermore, more severe stuttering was correlated with lower rCBF values in IFG. While this correlation is suggestive of a potential link between reduced activity in IFG and stuttering symptoms, it is important to emphasize the cross-sectional nature of this data, which precludes definitive conclusions about causality. It remains possible that reduced rCBF represents a primary deficit contributing to fluency disruption, a secondary effect of atypical neural organization, or an epiphenomenon unrelated to the core pathophysiology. Alm (2021) [13] reviews additional evidence for the “metabolic theory” of stuttering, including several EEG studies that found decreased beta band (~13–20 Hz) power in people who stutter compared to controls [11,14,15,16], similar to what is seen during mild hypoxia and hyperventilation and consistent with reduced rCBF at rest. Furthermore, the decrease in beta power is remarkably consistent within the stuttering groups; Alm states that this homogeneity suggests that it may reflect a core aspect of stuttering.

### 3.2. Cellular Metabolism and ATP Production

Another theory about the pathophysiology of DS is that it may be a disorder related to an impairment of energy supply to neurons. Multiple brain imaging studies have shown that people who stutter have low brain activity in the left-sided cortical speech sites, mainly in the areas associated with language processing, as well as in areas associated with timing and coordination of motor function of speech production [16]. Since speech is a complex neuronal function with high energy demands, it requires the integration of multiple neuronal systems. Dysfunctional activation of these systems or regions may indicate an underlying neuronal energy deficiency. A study by Neef et al. [17] suggested that an important aspect of stuttering may be an impaired firing frequency of the speech motor system. The study was conducted with Transcranial Magnetic Stimulation (TMS), which showed that participants who stuttered had a limited activation of the primary motor cortex of the tongue immediately prior to initiating a speech sound, suggesting an acute effect of metabolic limitations [13]. Alternatively, Chow et al. [18] and Boley et al. [19] proposed that limitations in energy metabolism might affect childhood development, especially in periods of rapid use of energy, such as speech production. Regardless of the differences argued in these studies, the common finding between these studies is a limitation in neuronal energy supply, which can most likely have developmental and/or momentary effects on speech production.

### 3.3. Neuronal Activity, Connectivity, and Anatomy

By the end of the 1990s, after the advent of non-invasive technologies for measuring brain activity, such as positron emission tomography (PET) and functional magnetic resonance imaging (fMRI), various neuroimaging studies focused on measuring and comparing brain activity during speech in people who stutter (PWS). One common finding from these PET and fMRI studies is the anomalous cortical lateralization during speech in PWS, with decreased left hemisphere activation and/or increased right hemisphere activation compared to controls [20,21,22,23]. Other studies using magnetoencephalography (MEG) found right-hemisphere-dominant suppression of motor rhythms (which decrease during motor processing) in PWS, providing further evidence of over-reliance on right-hemisphere cortical mechanisms in stuttering [24]. Together these findings indicate that stuttering is accompanied by abnormally low activity in the left hemisphere regions responsible for the planning and execution of speech motor programs [25], a phenomenon that may constitute a key pathophysiological mechanism underlying the disorder.

MRI has also produced a wealth of information regarding neural structure and connectivity in PWS. Reduced thickness or area of left-hemisphere cortical regions involved in speech, such as the IFG and ventral motor and premotor cortices, has been consistently identified in children who stutter (e.g., Chang et al. 2008 [26], Beal et al. 2012 [27]), though studies of adults who stutter and longitudinal studies suggest that the IFG difference may decrease with age [28,29]. Other studies using diffusion-weighted imaging (DWI) also found reduced white matter integrity in key left hemisphere cortico-cortical tracts, including the frontal aslant tract [30], which connects the medial supplementary motor areas with IFG, and the arcuate fasciculus [31], which connects auditory cortical areas with frontal cortical areas involved in speech planning. In 2014, Cai et al. [32] found negative correlations between stuttering severity in adults and tract strength in several left hemisphere speech network tracts, further supporting the lateralization atypicality view dating back to at least 1927 [33], in which left hemisphere deficiencies force right hemisphere compensations. Modern theoretical treatments focus on the cortico-basal ganglia-thalamo-cortical loop, which is thought to be heavily involved in feedforward control of speech (i.e., the readout of stored speech motor programs [34]), as a prime candidate for the underlying pathophysiology of stuttering [35,36], with this feedforward control deficit forcing increased use of the right-lateralized feedback control network [25,36]. This view is further supported by findings of impaired connectivity in children who stutter between cortical and subcortical components of the loop, in particular the putamen and thalamus [31,37].

The findings from both functional and structural imaging portray a consistent picture of altered neural processing in stuttering. The observed patterns of atypical brain activity during speech, particularly the reduced left-hemisphere involvement, appear to be associated with underlying structural differences and compromised connectivity within key speech networks. This reflects a robust correlation between functional and anatomical factors in people who stutter. While these findings highlight a distinct neurobiological signature, current cross-sectional data cannot definitively establish a causal direction; thus, these differences may represent either the underlying drivers of the disorder or the neuroplastic result of long-term stuttering behavior.

### 3.4. Genetics and Impact (Astrocyte Function)

N-acetylglucosamine-1-phosphate transferase subunits α and β (GNPTAB) are genes that encode a component necessary for proper lysosomal trafficking. In 2019, a groundbreaking discovery in the genetics of stuttering was made in a study by Tae-Un Han et al. [38], which directly linked mutations in the GNPTAB gene with stuttering. It is important to contextualize this finding: such mutations have been identified in a subset of individuals with familial stuttering and are not representative of the broader, non-familial stuttering population. When these human mutations were introduced into genetically engineered mice, similar vocalization deficits were identified. Subsequent immunohistochemistry of rodent brain tissues revealed a marked decrease in astrocyte dysfunction as a plausible biological mechanism in this specific genetic subtype. While this provides a compelling model for one pathophysiological pathway to stuttering, it remains to be determined whether astrocyte pathology is a feature of the more common, non-syndromic forms of the disorder. Further research is needed to translate these findings from rare genetic variants to the general population of people who stutter. At the same time, it has been determined that N-Acetylglucosamine-1-Phosphotransferase, in the γ subunit (GNPTG), encodes a protein subunit that combines with the product of the GNPTAB gene to form a functional phosphotransferase enzyme involved in the process of targeting lysosomal enzymes for degradation [39]. Mutations in GNPTG could contribute to lysosomal issues, which could disrupt neural networks involved in speech and motor planning.

Finally, the N-Acetylglucosamine-1-Phosphodiester Alpha-N-Acetylglucosaminidase (NAGPA) gene functions downstream to GNPTAB and GNPTG [39], ensuring proper enzyme activation and targeting. When mutated, incomplete lysosomal enzyme tagging occurs, translating to further lysosomal dysfunction. This could explain the impaired maintenance of neural pathways necessary for fluent speech.

In summary, the mutation in any of these three genes could lead to lysosomal dysfunction, altering cellular homeostasis. In the context of neural tissue, such disruptions could affect motor control pathways essential in speech rhythm and fluency.

### 3.5. Birth Trauma and Brain Damage in Stuttering

For many years, both genetic and non-genetic factors have been studied in hopes of finding the cause of stuttering. Given that persistent stuttering is a predominantly male disorder and knowing that at least half of all stuttering cases are not inherited, Drayna et al. [40] explored a non-genetic factor that primarily affects males: perinatal hypoxia. Accordingly, Poulos and Webster [41] conducted a study with 171 participants (ages 14–69 years) who stuttered and found that individuals without a familial history of stuttering have a significantly higher prevalence of birth or developmental trauma, suggesting a non-genetic pathway involving early-life neurological insults [4]. Notably, spontaneous recovery from stuttering is common in young children, with reports stating 80% or more of cases resulting in complete resolution. The limited published literature suggests that recovery involves normalization of connectivity deficits in subcortical white matter regions such as the corticospinal tract, superior longitudinal fasciculus, and arcuate fasciculus, enabling fluent speech [42]. In summary, this data suggests that early life brain injury and hypoxia may be related to the onset of DS and that recovery is possibly influenced by neuroplasticity and re-establishment of neuronal connections.

### 3.6. Mood Disorders in Stuttering

Stuttering has been linked to a higher risk of developing social anxiety, a twofold increase in mood disorders, and a threefold increase in personality disorders, most likely secondary to the cumulative negative impact of DS on patients’ lives [6]. A Chinese study from 2017 was able to observe higher brain activity regions associated with states of anxiety during a syllable repetition task in adults who stutter [43]. There is also suggestive evidence that people who stutter have a greater tendency to focus their attention on negative thoughts when entering a (feared) social situation, unleashing symptoms of anxiety, as well as recurring negative emotions such as guilt, shame, embarrassment, fear, helplessness, and frustration [5].

Neuroscientific findings suggest that these emotional vulnerabilities may have a neurobiological basis. Structural differences have been identified in limbic brain regions responsible for emotional regulation and reward processing, including the nucleus accumbens and amygdala [42], which could further contribute to emotional symptomatology. This, in turn, may reinforce the cycle of negative social experiences and emotional distress.

Furthermore, stuttering has been associated with lower quality of life and occupational and educational barriers [6], further exacerbating emotional challenges.

## 4. Transcranial Photobiomodulation

Before examining the potential application of t-PBM to DS, it is essential to review its established mechanisms of action. The following subsections summarize findings from preclinical studies, animal models, and human clinical trials. Where mechanisms are well characterized in cellular or animal systems but have not been conclusively demonstrated in humans, this distinction is explicitly noted. Subsequently, we will explore how these mechanisms might hypothetically address the pathophysiological deficits identified in DS.

### 4.1. Cerebral Blood Flow

The effects of t-PBM on cerebral blood flow have been a topic of interest for many years. In 2009, a study on a group of patients with major depressive disorder reported a significant increase in the regional cerebral blood flow following therapy with near-infrared and red-light therapy; no specific mechanism for these effects was established [44]. More recent studies have been able to prove that PBM can lead to enhanced rCBF, presumably via metabolic effects that elevate nitric oxide (NO) levels in the brain, which will ultimately lead to widening of blood vessel diameter. Uozumi et al. proposed that following brain PBM therapy, rCBF is elevated by 30% in the illuminated hemisphere and 19% in the opposite hemisphere, accompanied by a 50% rise in cortical NO concentration [45].

Beyond its direct hemodynamic effects, t-PBM engages NO as a mediator with broader implications for vascular and lymphatic function. When NO photodissociates from cytochrome C oxidase (CCO), it restores mitochondrial respiration while simultaneously increasing local NO bioavailability [45]. Emerging evidence suggests that NO-mediated mechanisms may extend to the lymphatic and glymphatic systems. NO also induces relaxation of lymphatic endothelium via the soluble guanylate cyclase/protein kinase G pathway, facilitating diastolic filling and contractility of meningeal lymphatic vessels, which is essential for the drainage of metabolic waste and toxins from the brain [46]. Furthermore, by modulating endothelial permeability and lymphatic contractility, t-PBM may enhance glymphatic clearance, particularly during sleep when this system is naturally activated [47]. These systemic effects raise the possibility that t-PBM’s therapeutic benefits in deeper cortical layers may involve not only direct photon action on neurons and astrocytes but also indirect mechanisms mediated by improved waste clearance and vascular dynamics. While these pathways are increasingly characterized in preclinical models, their relevance to human applications, including DS, warrants further investigation.

### 4.2. Cellular Activity and ATP Production

t-PBM therapy has been demonstrated to enhance neuronal activity in vitro and in animal models, primarily through mechanisms involving increased cellular ATP production [45]. The proposed mechanism is as follows: hypoxia or cellular damage results in NO impeding the enzymatic activity of CCO. Photons delivered during red and NIR light therapy are absorbed by this enzyme, which is thought to lead to NO dissociation, increased mitochondrial membrane potential, enhanced oxygen consumption and glucose metabolism, and finally, increased mitochondrial ATP. Similarly, research in embolic stroke models of rabbits revealed that transcranial laser therapy, even if only one session, significantly boosted cortical ATP concentrations [45]. Clinically, this was supported by a study where t-PBM at different wavelengths was able to affect neural oscillations, resulting in increased alpha, beta, and gamma waves on EEG, reflecting increased neuronal activity [48].

### 4.3. Neuronal Activity and Connectivity

Clinical studies in patient populations, such as those with chronic stroke or Alzheimer’s disease, have reported that t-PBM can influence functional connectivity, suggesting a potential for network-level modulation [49]. These observations are supported by a substantial body of preclinical evidence demonstrating that PBM can stimulate synaptogenesis, neurogenesis, and neuroplasticity in animal models and cell cultures [50]. The molecular and cellular mechanisms underlying these effects are increasingly well characterized and provide a plausible explanation for the network-level changes observed in human studies. By modulating voltage-gated calcium channels, PBM can trigger signaling pathways (i.e., the CaMKII cascade) essential in synaptogenesis by facilitating recruitment and clustering of synaptic proteins. It can also modulate intracellular signaling pathways (i.e., AKT/PI3K and MAPK/ERK) essential in neuronal growth, differentiation, and synaptic plasticity. PBM also upregulates brain-derived neurotrophic factor (BDNF) and activates tropomyosin receptor kinase B (TrkB), critical for neural stem cell differentiation [51]. Finally, the influence of PBM on potassium and sodium ion channels grants it the ability to stabilize membrane potentials, thus improving synaptic efficacy and enhancing neuroplasticity. Collectively, these processes reinforce the formation and adaptation of neural circuits [51].

The translation of these cellular effects to functional improvements in humans is an active area of investigation, and the precise mechanisms by which t-PBM modulates human neural networks remain to be fully elucidated.

### 4.4. Astrocyte Proliferation and Function

A 2021 in vitro study from Korea exposed cultured astrocytes to 660-nanometer LED light and reported that PBM enhances astrocyte proliferation under these experimental conditions [52]. This happens by increasing ATP production and signaling of controlled reactive oxygen species, which activates pathways such as the MAPK/ERK cascade, thus promoting cell cycle progression and increasing proliferation markers such as bromodeoxyuridine (BrdU) and Ki67, as well as an increase in the expression of Glial Fibrillar Acidic Protein (GFAP), a key structural protein in astrocytes. While this finding provides a valuable proof-of-concept for PBM’s effects on glial cells, it is essential to recognize that cultured astrocytes in a dish lack the complex microenvironment of the human brain, including interactions with neurons, microglia, and the vascular system. Whether these proliferative effects translate to the in vivo human brain and whether they would be beneficial in the context of stuttering-related pathology remains to be determined.

### 4.5. Brain Inflammation and Damage

The proposed neuroprotective effects of t-PBM are grounded in a well-characterized cascade observed in cellular and animal models. When NIR light photons are absorbed by CCO in the mitochondrial respiratory chain, this stimulates ATP production and activates downstream signaling pathways via a brief burst of reactive oxygen species (ROS). In preclinical models, this signaling has been shown to reduce overall oxidative stress by activating cellular antioxidant defenses and decreasing proinflammatory cytokines and neuroinflammation [44]. However, the extent to which this cascade is engaged in the human brain following transcranial irradiation, and whether it contributes to clinically meaningful neuroprotection, requires further investigation through human biomarker studies and clinical trials. On the other hand, excessive ROS produced by trauma activates NF-κB signaling pathways, which are blocked by t-PBM, thereby mitigating inflammatory reactions [45]. Moreover, t-PBM upregulates neurotrophins, such as brain-derived neurotrophic factor, which can stimulate the processes of synaptogenesis (formation of new connections between existing neurons), neurogenesis (formation of new neurons from neural stem cells), and neuroplasticity, thus helping the brain to heal itself [50].

The NIR photons are absorbed by CCO in the mitochondrial respiratory chain. This mitochondrial stimulation increases production of ATP but also activates signaling pathways by a brief burst of ROS. This signaling activates antioxidant defenses, reducing overall oxidative stress. Proinflammatory cytokines and neuroinflammation are reduced. Neurotrophins such as brain-derived neurotrophic factor are upregulated, which in turn activates synaptogenesis (formation of new connections between existing neurons) and neurogenesis (formation of new neurons from neural stem cells).

### 4.6. Impact on Mood

In the last decade, PBM therapy has emerged as a promising treatment for mood disorders (such as depression and anxiety) through its various effects on the nervous system. As mentioned already, PBM affects ATP production and cerebral blood flow, boosting cellular energy, supporting brain function, and optimizing cognitive performance. At the same time, PBM has been shown to modulate neurotransmitters such as serotonin and NO [53], both of which play essential roles in mood regulation. Via multiple pathways and mechanisms, PBM has been suggested to be effective for the reduction of anxiety and depressive symptoms [53].

## 5. Discussion: Synthesis of Evidence and Hypothesis Generation

In this review, we have synthesized the evidence-based neurobiological foundations of DS and the known mechanisms of t-PBM. In the following sections, we transition to hypothesized generation, where we integrate these two domains to propose how t-PBM might specifically modulate the dysfunctional circuits associated with stuttering. While the underlying neurophysiology is well documented, the clinical application to DS remains a theoretical framework that requires rigorous empirical validation. To the best of our knowledge and after an extensive search in different databases, including PubMed, Google Scholar, Scielo, Web of Science, Sage Journals, Scopus, and Clinicaltrials.gov, there are no clinical studies currently investigating the effect of t-PBM on DS. However, based on existing literature, we hypothesize that t-PBM could offer a novel and effective treatment for DS, addressing its proposed underlying pathophysiological mechanisms. Before discussing these applications, it is important to note that the findings reviewed herein are primarily derived from the healthy subjects and patients with a variety of neurological and psychiatric disorders; currently, no direct evidence exists for subjects affected by DS. While it is reasonable to extrapolate these fundamental neurophysiological effects to the mechanisms of stuttering, we recognize this lack of disorder-specific data as a significant limitation that necessitates a cautious interpretation of its translation potential.

### 5.1. Hypothesized Impact on Cerebral Blood Flow

Research has shown an association between stuttering and reduced rCBF, supported by evidence from EEG and imaging studies, which also suggest an inverse correlation between the rCBF deficiency and stuttering severity. Photobiomodulation has been shown to enhance rCBF by increasing NO levels and blood vessel dilation, with research reporting significant rises in rCBF in the illuminated hemisphere and the opposite hemisphere. We hypothesize that targeted application to left-hemisphere speech regions (such as IFG) could ameliorate focal hypoperfusion documented in individuals who stutter. Whether such hemodynamic changes would translate into improved speech fluency is an empirical question that requires direct investigation; the hypothesis presented here posits a mechanism by which this could occur.

### 5.2. Hypothesized Modulation of Cellular Metabolism and ATP Production

DS may stem from impaired neuronal energy supply, with studies highlighting low activity in left-sided cortical speech areas, impaired speech motor system activation, and metabolic limitations affecting speech production and development. t-PBM therapy may enhance neuronal activity by increasing cellular ATP production through mechanisms such as NO dissociation and improved mitochondrial function, with studies showing boosted cortical ATP levels and increased neural activity reflected by increased alpha, beta, and gamma waves. If these metabolic effects translate to the speech-motor regions of individuals who stutter, they could potentially address the energy deficiency hypothesized to underlie DS. However, direct evidence linking t-PBM-induced metabolic changes to speech fluency improvements is currently lacking and represents a critical target for future research.

### 5.3. Potential Effects on Neuronal Activity and Connectivity

The pathophysiology of stuttering includes altered neuronal anatomy, activity, and connectivity, such as decreased left hemisphere activation and altered structure in regions critical for speech production and motor control. PBM has been observed to promote synaptogenesis and neurogenesis and promotes neuroplasticity as well as neuromodulating properties, which could potentially improve altered neural circuits implicated in stuttering. 

### 5.4. Proposed Role: Astrocyte Proliferation and Function

In vitro studies have demonstrated that PBM can enhance astrocyte proliferation and function, with proposed mechanisms including increased ATP production and activation of the MAPK/ERK cascade, which promotes cell cycle progression. While this provides a plausible biological link to GNPTAB-related astrocyte deficits observed in a subset of familial stuttering, it is critical to note that these are cellular-level findings that have not yet been replicated in individuals who stutter. Whether t-PBM can rescue astrocyte pathology in the human brain remains a compelling but untested hypothesis.

### 5.5. Plausible Impact of Birth Trauma and Brain Damage

Some studies suggest that DS may be linked to early-life brain injury and hypoxia, highlighting higher birth trauma rates in non-familial cases and suggesting that recovery, common in children, involves neuroplasticity and neuronal connection reestablishment. Although direct clinical data on t-PBM’s effect on birth trauma and brain damage in humans are limited, a substantial body of preclinical evidence-derived from animal models of stroke, traumatic brain injury, and hypoxia-suggests that PBM promotes brain healing through multiple mechanisms, including enhanced ATP production, reduced oxidative stress and inflammation, modulation of harmful signaling pathways (e.g., NF-kB), and upregulation of neurotrophins that support neurogenesis, synaptogenesis, and neuroplasticity ([44,45,50]). The extrapolation of these findings to human infants or adults with perinatal brain injury remains speculative, and translational studies are urgently needed to determine whether these neurorestorative effects are achievable and clinically meaningful in humans.

### 5.6. Impact on Mood (Depression and Anxiety)

Stuttering has been strongly associated with anxiety and depressive symptoms. PBM has demonstrated ameliorating effects on anxiety and depression through improving cellular function, blood flow, and modulation of neurotransmitters like serotonin and NO. This raises the hypothetical possibility that t-PBM could confer secondary mood-related benefits in individuals who stutter, potentially addressing the psychological burden of the disorder. However, this remains speculative and requires targeted investigation in the DS population.

### 5.7. Specificity of Effect (Targeting a Vulnerable System)

The effect of t-PBM on disorders with symptoms affecting speech, language, and hearing has been previously evaluated, with promising results and a high safety and tolerability profile [54]. A case report on the therapeutic effects of t-PBM plus speech-language therapy versus speech-language therapy alone in a stroke patient with aphasia showed significant improvement in rate of speech and utterance length after adding t-PBM to the treatment regimen [55]. Similarly, a 2019 report on the use of t-PBM in three children with Down syndrome indicates an improvement in verbal fluency after two weekly sessions of t-PBM for four continuous weeks [56]. In studies with children with autism spectrum disorder, treatment with near-infrared light demonstrated an increase in communication skills and a reduction in the Social Communication subscale in the Social Responsiveness Scale 2nd Edition (SRS-2) [57,58]. These findings could serve as initial support for our hypothesis on the use of t-PBM for DS.

The specificity of t-PBM’s therapeutic action in DS is likely mediated by three convergent factors: topographical targeting, state-dependent modulation, and the energetic demands of the speech-motor system.

First, t-PBM permits regional anatomical targeting rather than systemic administration. By concentrating irradiance on the left-hemisphere speech nodes (specifically the IFG and adjacent premotor cortices), the intervention delivers metabolic support directly to the structural nodes where functional deficits, such as reduced rCBF and impaired glucose metabolism, have been empirically documented [11,12]. Thus, clinical specificity is achieved through the focal delivery of photons to dysfunctional circuits within the speech-motor loop.

Second, the speech-motor network is characterized by a high energetic strain. Fluent speech production requires the rapid, millisecond-level coordination of multiple muscles at high speeds, imposing a high energetic burden on the underlying neural circuits [13]. This system has been demonstrated to operate with a reduced metabolic efficiency in individuals with DS, as evidenced by findings of hypoperfusion in left-hemisphere speech regions [11,12] and impaired activation of the speech motor cortex prior to speech initiation [17]. Consequently, interventions that enhance metabolic support or vascular perfusion (such as t-PBM, which increases ATP production and rCBF) may lower the functional threshold for fluent speech production in a compromised system while producing no discernible effect in healthy systems operating. This interpretation is consistent with observations that t-PBM in healthy controls often yields minimal cognitive enhancement, whereas similar interventions in clinical populations with documented metabolic or vascular deficits can produce meaningful improvements [49,50]. Thus, the apparent “specificity” of t-PBM for stuttering may not reflect a disorder-selective molecular mechanism but rather the targeted application of a general metabolic enhancer to a neural system with uniquely high and unmet energy demands.

Finally, it is important to acknowledge that t-PBM’s effects are unlikely to be entirely confined to targeted regions; some degree of network-wide modulation is inevitable. However, if the core deficit in DS lies within a specific node or circuit of the speech network, even partial restoration of function may yield benefits for the emergent property of fluency. Future studies combining t-PBM with functional neuroimaging will be essential to test this hypothesis by examining whether fluency improvements correlate with normalization of activity in targeted regions and whether such effects propagate through the broader speech network.

### 5.8. Treatment Parameters: Current Knowledge and Knowledge Gaps

A fundamental challenge in translating the t-PBM hypothesis into clinical trials is the absence of empirically derived treatment parameters for developmental stuttering. At present, no studies have investigated t-PBM in this population, and therefore any discussion of optimal wavelength, power density, treatment location, session duration, frequency, or total number of sessions is necessarily speculative. However, the broader t-PBM literature provides a foundation from which initial parameters can be extrapolated for proof-of-concept studies while emphasizing the critical need for systematic dose-finding research.

#### 5.8.1. Wavelength

t-PBM typically utilizes red (600–700 nm) or near-infrared (NIR; 700–1100 nm) light, with NIR wavelengths penetrating deeper into cortical tissue due to reduced scattering and absorption by chromophores such as hemoglobin and water [14,45]. Most transcranial applications employ NIR wavelengths in the range of 800–1100 nm, with 810 nm and 1064 nm being the most commonly studied [44,59,60]. For targeting left-hemisphere cortical speech regions—particularly the inferior frontal gyrus (IFG) and adjacent premotor areas—a wavelength in the NIR range (e.g., 810 nm or 1064 nm) is likely necessary to achieve sufficient photon penetration through scalp, skull, and meninges to reach cortical tissue.

#### 5.8.2. Treatment Location and Targeting

Based on the pathophysiology reviewed in Section 3, the primary target for t-PBM in DS should be left-hemisphere speech regions, with particular emphasis on the inferior frontal gyrus (Broca’s area) and ventral premotor cortex, where reduced rCBF and metabolic activity have been most consistently documented [11,12]. Secondary targets may include the supplementary motor area and sensorimotor cortex, given their roles in speech motor planning and execution [25,34]. For initial investigations, we propose a focused application to the left IFG, using either a single large emitter or an array of smaller emitters positioned over the F5/F7 region according to the 10–20 EEG coordinate system, which approximates the location of Broca’s area.

#### 5.8.3. Power Density and Fluence

Effective t-PBM requires sufficient radiant exposure (fluence) at the cortical surface. However, due to attenuation by the scalp and skull, the fluence delivered to the skin surface must be substantially higher than the desired cortical dose. Human cadaver and modeling studies suggest that at 810 nm, approximately 1–2% of surface irradiance reaches the cortical surface [45,61]. Therefore, to achieve a cortical fluence in the range shown to modulate neuronal activity in preclinical studies (approximately 1–10 J/cm^2^), surface fluences of 50–100 J/cm^2^ (sometimes more) may be required. Power densities at the scalp typically range from 10 to 500 mW/cm^2^, with higher power densities enabling shorter treatment sessions [44,59]. For initial DS studies, we suggest a surface irradiance of 100–250 mW/cm^2^, delivering a total fluence of 60–90 J/cm^2^ per session, consistent with parameters used in depression and stroke trials [44,59,60].

#### 5.8.4. Session Duration, Frequency, and Total Number of Sessions

Treatment protocols in the t-PBM literature vary widely. Session durations typically range from 4 to 20 min, with treatment courses spanning from single sessions to 12 weeks of daily or twice-weekly application [44,59,60]. For a chronic neurodevelopmental condition such as DS, a course of multiple sessions is likely necessary to induce sustained neuroplastic changes. We propose an initial protocol of 2–3 sessions per week for 4–8 weeks (total 8–24 sessions), with each session lasting 10–20 min. This aligns with protocols that have shown durable effects in depression and TBI [44,60]. However, we emphasize that this is a starting point for investigation; systematic dose-finding studies comparing different frequencies, durations, and total session numbers are urgently needed.

#### 5.8.5. General Safety Profile

In terms of safety, studies have proven t-PBM to be a safe and highly tolerable therapy. Three large randomized clinical trials (NEST-1, NEST-2, and NEST-3) evaluated the safety of t-PBM in a sample of 1410 stroke patients, concluding that no significant difference in the rate of adverse effects was observed between the group receiving the light therapy vs. placebo [59]. Similarly, two uncontrolled studies using 1 and 6 sessions of t-PBM reported that no severe side effects were associated with the treatment [60]. When reported, the most common side effects have been mild transitory headaches, insomnia, and erythema at the site of application, with a minimal risk for not significant thermal burns [61].

#### 5.8.6. Safety Considerations in Pediatric Populations

Given that DS onset occurs in early childhood and many individuals who stutter are minors, pediatric safety considerations are paramount. t-PBM has been used safely in children with autism spectrum disorder and Down syndrome, with no reported serious adverse events [56,57,58]. However, systematic safety data in pediatric populations remain limited. Parameters must be adjusted for the thinner skull and greater scalp transmission in children to avoid excessive cortical fluence. Initial pediatric studies should employ conservative parameters (lower power density, shorter sessions) and prioritize safety monitoring, including assessment of thermal effects, ocular safety, and neurodevelopmental outcomes.

### 5.9. Limitations and Counter-Evidence

While the preceding sections have outlined a hypothesis-driven rationale for t-PBM in developmental stuttering, it is essential to acknowledge the limitations of this approach and consider counter-evidence from the broader neuromodulation literature.

t-PBM may face physical limitations in penetrating deep subcortical structures implicated in DS. Monte Carlo simulation studies have demonstrated that while 810 nm light can reach superficial cortical regions up to 3–4 cm beneath the scalp, penetration is not uniform across all brain regions [62]. Critically, light from transcranial devices does not reach in sufficient quantities to elicit direct photochemical effects in deeper subcortical structures that may be involved in the pathophysiology of DS (such as the basal ganglia). However, its capacity to modulate superficial cortical targets with documented deficits—and potentially influence broader speech networks through connectivity-based effects—provides a plausible mechanism for therapeutic benefit.

The mixed outcomes of other neuromodulation approaches in stuttering serve as a cautionary tale, highlighting the importance of rigorous trial design, parameter optimization, and realistic expectations regarding effect sizes and durability. These considerations are incorporated into the research recommendations that follow.

Other preoccupations of t-PBM (and other neuromodulation) are related to the lasting effects of therapy once the number of sessions is achieved, with some reports of other neuropsychiatric disorders requiring perpetual non-invasive therapy to retain the desired effects of therapy.

## 6. Future Directions

Future research directions on t-PBM’s potential therapeutic properties in stuttering should emphasize a multifaceted approach. First, a certain degree of caution is required to interpret studies linking CBF alterations to stuttering, as most are based on cross-sectional data [4,5,43]. The heterogeneous nature of stuttering complicates the application of cbf-related findings to its different subtypes and prevents these findings from being interpreted as evidence of causality. It is plausible that the observed metabolic and vascular anomalies are biomarkers of the ‘stuttering state’—such as increased effort or anxiety—rather than the ‘stuttering trait itself. At present, no empirical evidence supports the clinical use of t-PBM for DS; the following recommendations are intended to guide the rigorous investigation of this hypothesis. Longitudinal studies are needed to improve our understanding of the relationship between altered CBF and stuttering severity. That being said, the exploration of t-PBM as a non-invasive and novel therapeutic approach for stuttering should encompass its potential to increase CBF, targeting hypoperfusion-related mechanisms.

Second, preclinical studies could use established animal models of stuttering and hypoperfusion to investigate t-PBM’s effect on cerebral metabolism, plasticity, and connectivity. Studies that explore the effect of t-PBM on ATP production and mitochondrial function in neurons implicated in speech-motor control could provide insights into its efficacy. Other models mimicking DS could be used to determine optimal therapeutic parameters (wavelength, intensity, duration).

Third, clinical trials are necessary to evaluate the efficacy of t-PBM in treating people who stutter. A randomized, sham-controlled trial could be used to assess the impact of t-PBM on speech fluency and, to a lesser degree, on improved psychosocial well-being in the long term. To determine the effect of t-PBM on brain perfusion in stuttering humans, measures such as biomarker analysis and CBF measuring via fMRI could prove useful.

Moreover, due to the developmental nature of stuttering, evaluating the effects of t-PBM across different age groups, such as children and adults, is crucial. Developmental stage, spontaneous recovery, and brain maturation should be considered core variables in trial design, as neurophysiological plasticity, network consolidation, and treatment responsiveness may differ between pediatric and adult populations.

Finally, neuroimaging studies that utilize modalities such as fMRI and diffusion tensor imaging could be leveraged to identify the neuronal mechanisms underlying the effects of t-PBM in stuttering and provide valuable information on white matter connectivity and changes in neural activity within speech-related brain regions. Assessment of the durability of t-PBM treatment effects could be achieved by implementing long-term follow-up studies, as well as determining the optimal frequency of maintenance sessions.

In sum, t-PBM holds promise as a cost-effective, non-invasive intervention for stuttering; however, comprehensive research, including preclinical studies, clinical studies, clinical trials, biomarker analysis, and functional neuroimaging, is needed to fully evaluate its therapeutic potential.

## Data Availability

No new data were created or analyzed in this study. Data sharing is not applicable to this article.

## Abbreviations

The following abbreviations are used in this manuscript:

DS	Developmental stuttering
CBF	Cerebral blood flow
t-PBM	Transcranial photobiomodulation
rCBF	Regional cerebral blood flow
tDCS	Transcranial Direct Current Stimulation
rTMS	Repetitive Transcranial Magnetic Stimulation
DBS	Deep Brain Stimulation
IFG	Inferior frontal gyrus
SFG	Superior frontal gyrus
TMS	Transcranial Magnetic Stimulation
PET	Positive Emission Tomography
fMRI	Functional magnetic resonance imaging
PWS	People who stutter
MEG	Magnetoencephalography
CCO	Cytochrome C oxidase
BDNF	Brain-derived neurotrophic factor
TrkB	Tropomyosin receptor kinase B
LED	Light Emitting Diode
BrdU	Bromodeoxyuridine
GFAP	Glial Fibrillar Acidic Protein
ROS	Reactive oxygen species
NO	Nitric oxide
